# Edition of Prostaglandin E2 Receptors EP2 and EP4 by CRISPR/Cas9 Technology in Equine Adipose Mesenchymal Stem Cells

**DOI:** 10.3390/ani10061078

**Published:** 2020-06-23

**Authors:** Ana Carolina Furlanetto Mançanares, Joel Cabezas, José Manríquez, Vanessa Cristina de Oliveira, Yat Sen Wong Alvaro, Daniela Rojas, Felipe Navarrete Aguirre, Lleretny Rodriguez-Alvarez, Fidel Ovidio Castro

**Affiliations:** 1 Department of Animal Science, Faculty of Veterinary Science, Universidad de Concepción, Campus Chillan, Chillán 3780000, Chile; joecabezas@udec.cl (J.C.); jmanriquez@udec.cl (J.M.); ywong@udec.cl (Y.S.W.A.); fenavarr@udec.cl (F.N.A.); llrodriguez@udec.cl (L.R.-A.); 2 Department of Veterinary Medicine, Faculty of Animal Science and Food Engineering, University of São Paulo, Pirassununga, São Paulo 13630-000, Brazil; van.oliveira@usp.br; 3 Department of Animal Pathology, Faculty of Veterinary Sciences, Universidad de Concepción, Campus Chillan, Chillán 3780000, Chile; drojasm@udec.cl

**Keywords:** CRISPR/cas9, prostaglandin E2, adipose mesenchymal stem cell, equine

## Abstract

**Simple Summary:**

Prostaglandin E2 plays a regulatory role in MSC self-renewal, and PGE2 receptor-mediated signalling is involved in cell proliferation and migration. The aim of this work was to establish a genetically modified equine cellular model in which receptor genes EP2 and EP4 were edited by the CRISPR/Cas9 system. We successfully targeted EP2 and EP4 receptors in horse adipose MSCs using the CRISPR/Cas9 system. This targeting did not affect the surface marker phenotype of the adipose MSCs generated. Gene edition significantly lowered the expression of each of the targeted receptors and affected the early migration ability of the edited MSCs. This opens the possibility of using these mutant cell lines as a model system to elucidate the role of EP2 and EP4 in MSCs and, particularly, as therapeutic tools in equine regenerative medicine.

**Abstract:**

In mesenchymal stem cells (MSCs), it has been reported that prostaglandin E2 (PGE2) stimulation of EP2 and EP4 receptors triggers processes such as migration, self-renewal, survival, and proliferation, and their activation is involved in homing. The aim of this work was to establish a genetically modified adipose (aMSC) model in which receptor genes EP2 and EP4 were edited separately using the CRISPR/Cas9 system. After edition, the genes were evaluated as to if the expression of MSC surface markers was affected, as well as the migration capacity in vitro of the generated cells. Adipose MSCs were obtained from Chilean breed horses and cultured in DMEM High Glucose with 10% fetal bovine serum (FBS). sgRNA were cloned into a linearized LentiCRISPRv2GFP vector and transfected into HEK293FT cells for producing viral particles that were used to transduce aMSCs. GFP-expressing cells were separated by sorting to obtain individual clones. Genomic DNA was amplified, and the site-directed mutation frequency was assessed by T7E1, followed by Sanger sequencing. We selected 11 clones of EP2 and 10 clones of EP4, and by Sanger sequencing we confirmed 1 clone knock-out to aMSC/EP2 and one heterozygous mutant clone of aMSC/EP4. Both edited cells had decreased expression of EP2 and EP4 receptors when compared to the wild type, and the edition of EP2 and EP4 did not affect the expression of MSC surface markers, showing the same pattern in filling the scratch. We can conclude that the edition of these receptors in aMSCs does not affect their surface marker phenotype and migration ability when compared to wild-type cells.

## 1. Introduction 

Prostaglandin E2 (PGE2) is an important mediator of several physiological processes, such as uterine contraction, cervix softening, fever induction, muscle relaxation, and vasodilation [1]. As many other mediators of cellular responses, PGE2 is multifunctional and plays an important role in limiting the inflammatory response by acting distinctly through one of four receptors, designated EP1, EP2, EP3, and EP4, which are encoded by separate genes; *PTGER1* to *PTGER4*, respectively [2,3]. 

The EP2 and EP4 receptors are members of the steroidal receptor family coupled to the G protein [4]. Their respective ligand, PGE2, is one of the predominant metabolites of arachidonic acid derivatives [5]. These receptors are involved in the increment of intracellular levels of cyclic adenosine 5’-monophosphate (cAMP) under PGE2 stimuli [6]. The expression of EP receptors and PGE2 is ubiquitous on the surface of cells in most systems and organs. This can explain the versatility of PGE2 effects in vivo, implicated in reproductive processes [7,8], cell proliferation, apoptosis, angiogenesis, inflammation, and immunological processes [9,10,11]. 

In mesenchymal stem cells (MSCs), it has been reported that stimulation of EP2 and EP4 receptors triggers migration, and are thus potentially involved in the homing ability of such cells [10,12,13]. Also, PGE2 helps to maintain features such as self-renewal [1], survival, and proliferation [14]. Furthermore, PGE2 has been indicated as one of the key factors of immunomodulation in equine [15] and human MSCs [16,17].

The use of gene knockout can contribute to the understanding of the potential physiology and pathophysiology of prostanoid receptors. The CRISPR/Cas9 (clustered regularly interspaced short palindromic repeat) system could be a useful tool for this purpose, and it has been proven to be an easier and more advantageous method compared to other genetic editing techniques, such as TALEN and Zinc Finger [18,19,20], both in animal models [20,21,22] and in human and veterinary medicine [23,24].

In our study, we separately edited the EP2 and EP4 receptor genes by CRISPR/Cas9 technology in MSCs derived from equine adipose tissue (aMSC) and evaluated the implication of PGE2 on the surface marker phenotype, cell migration, and the homing process.

## 2. Materials and Methods

### 2.1. Cell Culture

aMSCs were obtained from three Chilean breed horses and processed using previously described methods [25,26]. In brief, the adipose tissue was minced and digested for 2 h in 1 mg/mL collagenase-A (Sigma-Aldrich, St Louis, MO, USA) with gentle shaking in 15 mL sterile conical tubes in a water bath at 39 °C. Undigested tissue and clumps were allowed to settle down, and the supernatant was carefully removed and seeded in 60 mm sterile Petri dishes. 

The aMSCs and cells of the human embryonic kidney 293T cell line (Cat No. R70007, Invitrogen, Waltham, MA, USA) were cultured in Dulbecco’s modified eagle’s medium high glucose (DMEM, high glucose, GlutaMAX™ Supplement, Cat No. 10566024, Gibco™, Invitrogen, Rockville, MD, USA) with 10% foetal calf serum (FCS), 1 mM sodium pyruvate, and 100 U/mL of penicillin/streptomycin at 39 °C in 5% CO_2_.

All experiments were approved by the Ethics Committee of the Faculty of Veterinary Sciences, University of Concepcion (13/04/17, Approval Number: CBE-15-2017).

### 2.2. CRISPR 

#### 2.2.1. CRISPR Design

Horse genomic DNA from EP2 and EP4 receptors was amplified with primers, described in Table S1, and submitted for DNA sequencing (outsourced to Macrogen, New York, NY, USA). The guide RNAs were designed and analyzed with CRISPOR (http://crispor.tefor.net) to target exon 1 of the equine genes *PTGER2* (sgRNA: TGGTGCTGGCTTCGTACGCG; PAM: CGG) and *PTGER4* (sgRNA: GGAGACGACCTTCTACACGT; PAM: TGG/sgRNA reverse complement: PAM: CCA; CCAACGTGTAGAAGGTCGTCTCC), position sequence 226 bp and 230 bp, respectively, and synthesized by Integrated DNA Technologies (IDT, Coralville, IA, USA).

#### 2.2.2. Cloning and Hybridisation of gRNA Oligonucleotides

**Figure 1:**
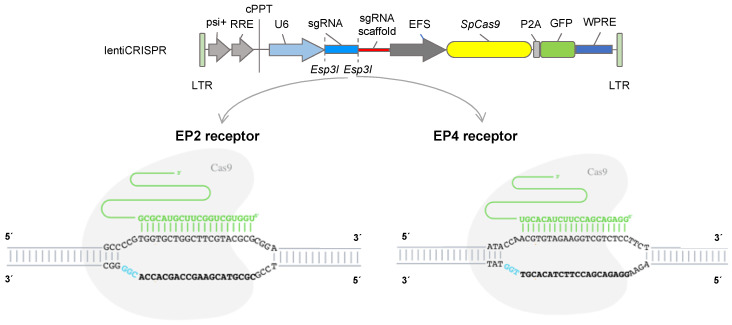
Upper panel: map of the LentiCRISPRv2GFP vector (Addgene plasmid #82416) encoding for Cas9 and GFP under the control of EFS promoter. The blue rectangle flanked by Esp3I sites is the cloning site for specific guide RNAs, located under the human U6 RNA polymerase III promoter. Lower panel: specific guide RNAS (sgRNA) in green with PAM sequence (light blue) targeting exon 1 for knock out of equine *PTGER2* (EP2 receptor) and *PTGER4* (EP4 receptor) genes.

Oligos were annealed with gRNA sequences and cloned into the digested LentiCRISPRv2GFP vector according to the protocol from the Zhang lab [27]. In brief, the oligos were resuspended at a concentration of 100 μM in ddH_2_O, and 1 μL each of the sense and antisense primers were added to a mixture of 6.5 μL water and 1 μL T4 ligation buffer and hybridised at 95 °C for 5 minutes, then cooled at room temperature for 2 h. The LentiCRISPRv2GFP vector (Figure 1; Addgene plasmid #82416) was linearized by Esp3I digestion (Cat. No ER0451, ThermoFisher, Waltham, USA), and 1 μL of the product of hybridisation was mixed with the linearized vector and ligated with 1 μL of T4 DNA ligase, 10× T4 DNA Ligase buffer (ThermoFisher, Waltham, MA, USA), and water for a total reaction volume of 10 μL. The mixture was incubated at 22 °C for 10 min and then at 65 °C for 10 min to inactivate the enzyme. The lentiviral vectors for EP2 and EP4 were transformed into chemically competent *E. coli* (Cat. No. K457501, ThermoFisher, Waltham, MA, USA), produced on a large scale and subjected to DNA maxiprep extraction (Cat. No.12162, QIAGEN Plasmid Maxi Kit, Qiagen, Hilden, Germany).

### 2.3. Lentivirus Production and Transfection 

Polyethylenimine (PEI; Cat. No. 408727, Sigma-Aldrich, St Louis, MO, USA) was used for the lentiviral transfection. A total of 6 × 10^6^ 293FT cells were plated in 100 mm diameter dishes one day before and allowed to reach 90% confluence on the day of transfection. Twelve micrograms of the respective lentiviral vectors and 8 μg of auxiliary packaging vectors, with the PEI reagent (1:1 ratio of DNA/PEI), were used in the transfection per plate. The cells were incubated overnight and cultured further, and the culture medium was collected after 24 h and 48 h, filtered, ultracentrifuged, and used for transduction. 

### 2.4. Transduction of aMSCs and Cell Clones

For transduction, 2 × 10^4^ aMSCs were cultured in 6-well plates and transduced with EP2ko or EP4ko viral particles in the presence of 8 ng/mL polybrene (Cat No. H9268, Sigma-Aldrich). After 48 h of transduction, the cells were sorted by means of the FACS-Aria II flow cytometer (BD BioSciences, Cockeysville, MD, USA) using cellular fluorescence. GFP-expressing cells were isolated by sorting one cell per well on 96-well plates and expanded to obtain individual clones. 

### 2.5. PCR and T7 Endonuclease I (T7EI) Cleavage Assays for Detection of Insertions and Deletions of Clones

For validation of clones, genomic DNA was extracted from clones of aMSC/EP2, aMSC/EP4, and aMSC-WT cells using the E.Z.N.A.^®^ Tissue DNA Kit (Cat. No. D3396-01, Omega Bio-Tek, Norcross, GA, USA). The PCR amplification was performed using Phusion High-Fidelity PCR Master Mix (Cat. No. F553L, ThermoFisher) with primers on-target (gRNA target site) for EP2 and EP4 (Table 1). PCR products were mixed with 2 μL of NEB Buffer 2.0 and water to make a total volume of 19 μL. The mixture was denatured and annealed to form heteroduplexes, followed by enzymatic digestion with 1 μL of T7EI (Cat. No. M0302S; New England Biolabs, Amherst, MA, USA) at 37 °C for 30 min. To analyze the DNA digestions, the products were resolved electrophoretically on a 2% agarose gel. 

### 2.6. Cloning and Analysis of Indel (Random Insertion or Deletion) Frequencies 

To identify the mutant alleles, the PCR products were cloned into the pUC57 vector. Both the PCR products and the vector were digested with the EcoRV restriction enzyme to generate blunt ends. The recombinant clones were confirmed by PCR followed by Sanger sequencing (outsourced to Macrogen) using M13 primers. After sequencing, the clones were analyzed by tracking indels by Inference of CRISPR Edits (ICE; Synthego, Redwood City, CA, USA; https://ice.synthego.com/#/).

### 2.7. RNA Isolation and Quantitative Real-Time Reverse Transcriptase PCR Analysis 

The total RNA from aMSC/WT, aMSC/EP2, and aMSC/EP4 was isolated with the E.Z.N.A.^®^ Total RNA Isolation Kit (Omega Bio-Tek), according to the manufacturer’s protocol. The RNA concentration and relative quality were assessed by spectrophotometry using a ratio of 260/280 as the criterion of purity in an Epoch Micro-Volume Spectrophotometer (BioTek, Winooski, VT, USA). The cDNA was synthesised in 500 ng of the total RNA using a high-capacity cDNA reverse transcription kit (Applied Biosystems, Foster City, CA, USA) according to the manufacturer’s instructions.

The qPCR reaction was performed in 10 μL using the KiCqStart^®^ (Saint Louis, MI, USA) SYBR^®^ (Saint Louis, MI, USA) Green qPCR ReadyMix™ with ROX™ (Sigma-Aldrich, Saint Louis, MI, USA). The cycling parameters for the qPCR reaction were as follows: 95 °C for 10 min; 40 cycles of 95 °C for 15 s, 58 °C for 30 s, and 72 °C for 30 s; and 72 °C for 2 min. The relative mRNA expression was calculated using the 2^−ΔΔCT^ method [28]. The aMSC-WT (wild-type) was used as a calibrator, and the geometric mean of two housekeeping genes (*ACTB* and *GAPDH*) was used as a normaliser. The primer sets and product size for real-time PCR are listed in Table 2. 

### 2.8. Immunocytochemical Analyses 

Immunocytochemical analysis was performed on cells to detect EP2 and EP4 receptor expression after gene edition. The cells were cultured in duplicate in 4-well chamber slides (Cat. No 154453, ThermoFisher), fixed in ice-cold methanol for 10 min at 4 °C, and washed with PBS 3 times for 5 min. Then, the cells were permeabilisated with 0.1% Triton X-100 solution for 10 min, washed 3 times with PBS, and blocked with 2% BSA in PBS for 1 h at room temperature. The cells were incubated with the primary antibodies (anti-EP2 Receptor, Cat. No. 101750, or anti-EP4 Receptor, Cat. No. 101775; 1:100 dilution, Cayman Chemical, Ann Arbor, MI, USA) at room temperature for 2 h, washed, and labelled with the secondary antibody (anti-rabbit Alexa Fluor 594, R37117; 1:300 dilution, Invitrogen) for 1 h. For nuclear counterstaining, the cells were washed 3 times with PBS, labeled with NucBlue™ Stain (Cat. No. R37605, ThermoFisher) for 5 min, and washed again. EP2 and EP4 peptide blocking was performed for negative controls (EP2 Receptor Blocking 175 Peptide, Cat. No. 301750; and EP4 Receptor [C-Term] Blocking Peptide, Cat. No. 301775, dilution 1:100, Cayman Chemical, Ann Arbor, MI, USA). Images were obtained with a laser scanning LSM780 confocal microscope (Carl Zeiss Microscopy GmbH, Oberkochen, Germany). Unedited wild-type aMSCs (aMSC-WT) were used as positive controls.

### 2.9. Cell Migration Assessed by Scratch Assay 

A wound healing scratch assay was used to evaluate cellular migration, as described previously [25]. The cells, aMSC-WT, aMSC-EP2, and aMSC-EP4, were cultured in 12-well plates until full confluency and exposed to 10 mg/mL of mitomycin C (Sigma-Aldrich) for to 2 h to avoid filling the gap by proliferation. The cell monolayer was scraped in a straight line to create a “scratch” with a 200 μL pipet tip and washed with PBS to remove the debris and to smooth the edge of the scratch. Then, the cells were incubated with culture medium supplemented with 10% FBS and 3 μM of PGE2 (Cayman Chemical). Serum-free cultured medium was used in the controls. The migration was evaluated as the average change in the width of the cell-free zone at time points 0, 24, 36, and 48 h (at 0 h, the full area is free of cells = 100%). The images were captured using an inverted EVOS FL fluorescence microscope (ThermoFisher), and the data were analysed with ImageJ software (https://imagej.net).

### 2.10. Expression MSC Surface Markers 

The surface markers of the MSCs (determined by accepted consensus), described by Dominici et al. [29], were analysed by conventional PCR. The PCR reaction was performed in 20 μL using Phusion High-Fidelity PCR Master Mix (Cat. No. F553L, ThermoFisher). The analyzed transcripts and the used primers are shown in Table 2 and were CD29, CD44, CD90, and CD166 for MSCs; CD45 for hematopoietic markers; and MHC I and MHC II for immune response, as previously described [30]. *ACTB* and *GAPDH* were used as reference genes. PCR products were analyzed on 1% agarose gel by electrophoresis.

### 2.11. Statistical Analysis

Analysis of variance (ANOVA) was used to test the statistical significance. For qPCR analysis, one-way ANOVA was used, with a Dunnett’s multiple comparisons post-hoc test. For the cell migration assay, two-way ANOVA with Tukey’s post-hoc test for multiple comparisons was performed. *p* < 0.05 was considered statistically significant. The results are expressed as the mean ± standard error of the mean (SEM). All data were analysed using the statistical package GraphPad Prism 8 (San Diego, CA, USA). 

## 3. Results

### 3.1. Transduction of aMSCs and Culture of Cellular Clones

**Figure 2:**
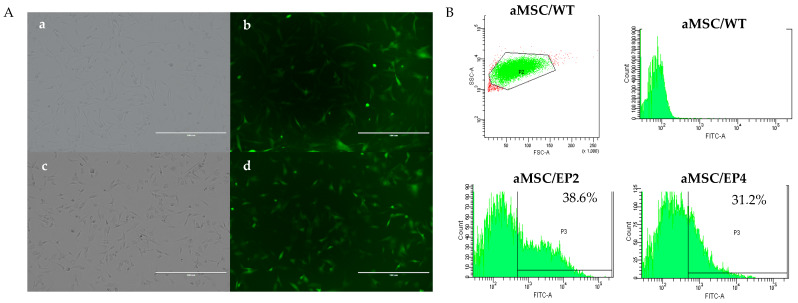
Evaluation of reporter GFP expression after transduction of MSC with lentiviral constructs carrying gRNA-Cas9 and GFP. The cells adhered to plastic and exhibited a fibroblast-like morphology. (**A**), photomicrographs of aMSC/EP2 and aMSC/EP4 with approximately 60% of confluence. (a, c, respectively), and with GFP-positive staining (b: aMSC/EP2), (d: aMSC/EP4 cells. (**B**), representative histograms and gating plots of GFP reporter cell populations analyzed: aMSC/WT, aMSC/EP2 and aMSC/EP4.

Viral particles were efficiently produced in HEK 293 cells (not shown) and were used to transduce aMSCs. The efficiency of transduction was monitored visually by eGFP expression in the aMSCs (Figure 2A) and was quantified by FACS. Positivity to GFP were: 38.6% (for EP2 cells) and 31.2% for EP4 (Figure 2B). The GFP-expressing cells were sorted and plated individually in 96-wells dishes and allowed to grow for 20 days until reaching confluence. At this point, cells were tripsinized, transferred to 12-well-plates, and cultured until confluence for further analysis. All cell lines adhered to the plastic and exhibited a fibroblast-like morphology, as shown in Figure 2A.

### 3.2. T7EI Test, Cloning, and Frequencies of Indels

**Figure 3:**
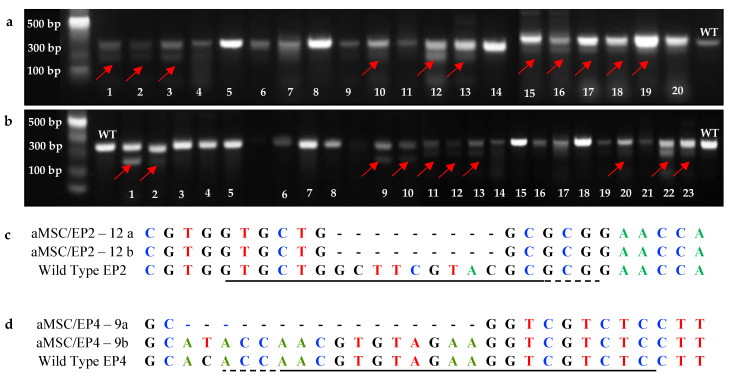
Mutation detection of EP2 and EP4 receptor gene by T7EI cleavage assay and sequences of alleles identified by Sanger sequencing. In (**a**), T7E1 digestion products analyzed by agarose gel electrophoresis for (**a**) aMSC/EP2, note mutations indicated by red arrows (1, 2, 3, 10, 12, 13, 15, 16, 17, 18 and 19). In (**b**) for aMSC/EP4 note mutations indicated by red arrows (numbers 1, 2, 9, 10, 11, 12, 13, 20, 22 and 23). In (**c** and **d**), the sequence of gRNA is shown in horizontal black underlined region, the PAM site is dotted region. In aMSC/EP2 (**c**) sequenced colonies 12a and 12b showed mutations with 9bp deletion (dotted). For aMSC/EP4 (**d**) sequenced colony 9a showed mutation with 10bp deletion after PAM (dotted) while sequenced colony 9b showed no changes and it is identical to wild-type alleles.

We further analyzed the actual cloning efficiency and details of GFP-positive clonal cells using the T7E1 assay. The DNA from individual clones was digested with the T7E1 enzyme, and the digestion products were analyzed by agarose gel electrophoresis. Cleavage identified 11 clones of aMSC/EP2 and 10 clones of aMSC/EP4 (Figure 3). Colonies from the clones were subjected to gRNA-specific PCR, and the amplification products were cloned and sequenced.

One out of the 11 aMSC/EP2 clones identified by T7EI digestion had a 9 bp deletion in both alleles and was considered a knock-out (KO) (9 bp deletion), with an indel frequency of 94% and KO score of 2. In aMSC/EP4, 10 clones were analysed, and 1 had a heterozygous mutation with 1 allele edited (10 bp deletion; Figure 3) and an indel frequency of 13% and KO score of 10. 

### 3.3. Immunocytochemistry and Gene Expression for EP2 and EP4 Receptors

**Figure 4:**
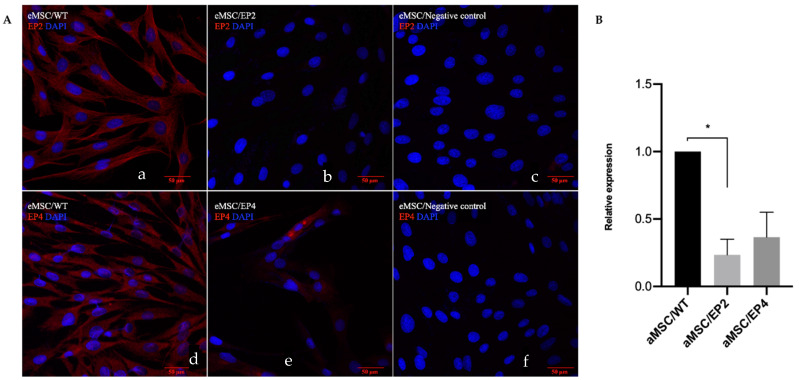
Evaluation of the presence of PGE2 receptors in edited aMSC. In (**A)**, expression of prostaglandin E2 type 2 receptor (EP2) (a) and EP4 (d) in aMSC/WT. Expression of EP2 in aMSC/EP2 (b); and type EP4 in aMSC/EP4 (e); negative controls (c, f). DAPI (blue) stains nuclei, EP2 and EP4 receptors are stained in red. In (**B)**, differences in relative amounts of mRNA expression for EP2 and EP4 receptors in aMSC. Data are expressed as relative expression regarding wild type (aMSC/WT). Asterisk indicates statistically significant differences with *p* < 0.05.

Immunocytochemical and qPCR analyzes were performed to validate the actual effect of the edition of the receptors on cells in vitro. EP2 and EP4 were localized in the cell membrane in the majority of aMSCs/WT (Figure 4A, a and d). The immune reaction of EP2 decreased in aMSCs/EP2 (Figure 4A, b) as well as the immune reaction of EP4 decreased in aMSCs/EP4 (Figure 4A, e). In the gene expression analysis, downregulation of *PTGER2* (4.3-fold, *p* < 0.04) was found in aMSCs/EP2, while the mRNA for *PTGER4* decreased 2.7-fold (*p* < 0.07) compared to the WT (Figure 4B). These results indicate that gene edition changed the pattern of expression of EP2 and EP4 receptors at both mRNA and protein levels.

### 3.4. MSC Surface Markers and MSC Migration

**Figure 5:**
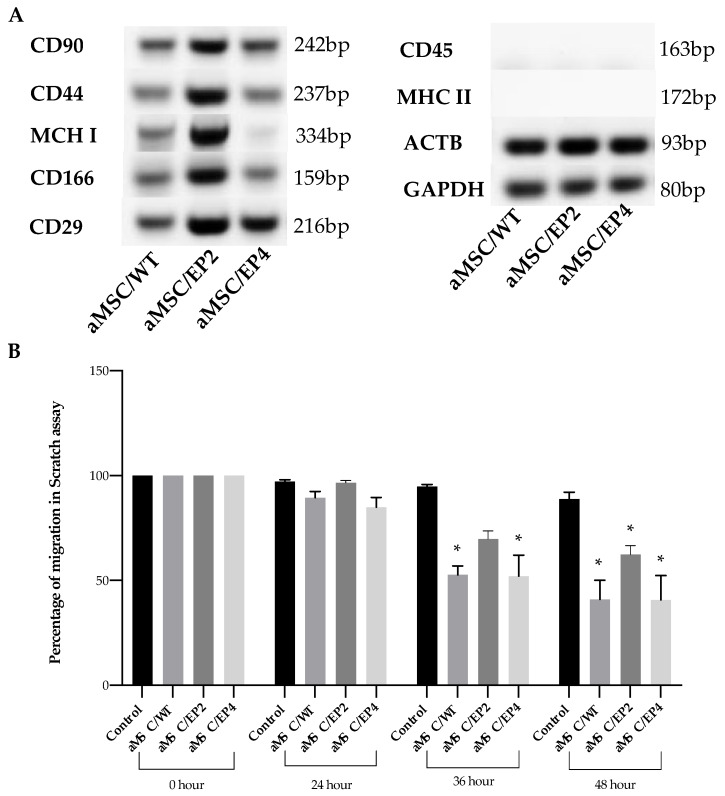
Effect of gene edition on the expression of surface markers in MSCs (**A**) and migration assay (**B**). In (**A**), gene expression pattern for referential positive CD90, CD44, MHCI, CD166, and CD29; and negative CD45 and MHCII horse mesenchymal cell markers. In (**B**), evaluation of the in vitro migration ability of manipulated aMSCs towards 3 μM PGE2. Columns represent the percentage of area uncovered by the cells at 0, 24, 36 and 48 h. Control: wild type cells attracted toward serum-free medium; WT: aMSC non genetically edited; EP2ko and EP4 ko; aMSC edited genetically for EP2 and EP4 receptor gene respectively. Asterisk indicates statistically significant differences between control and aMCS/WT, aMSC/EP2 and aMSC/EP4 (*p* < 0.05).

Next, we focused on whether gene edition of the two receptors influenced the expression of MSC surface markers in RT-qPCR assays and the migration ability of edited cells. The pattern of expression of the selected markers did not change among the edited and wild-type cells. All cells were positive for CD90, CD44, CD166, CD29, and MHCI and negative for CD45 and MHCII surface markers (Figure 5A). 

The scratch assay allowed for quantitative analysis of cell migration as a function of time. Twenty-four hours after creation of the scratch, there was significantly (*p* < 0.05) impaired migration of aMSCs/EP2 compared to the wild type and aMSCs/EP4; however, at 36 h, all cell types tested had similar filling of the wound area, except for the control group, without attractant (Figure 5B). 

## 4. Discussion 

MSCs have emerged as the most used stem cell type for clinical applications due to numerous advantages [31], including their ability to home damaged tissues, their ability to differentiate into various cell types, and their pleiotropic effects [32]. Their immunological properties, including anti-inflammatory, immunoregulatory, and immunosuppressive capacities, contribute to their potential role as immune tolerant agents [33,34].

In 2005, the U.S. Food and Drug Administration (FDA) reported that horses were the most appropriate animal model for the evaluation of clinical effects of MSC-based therapies for certain injuries in humans [35]. In horses, the vast majority of reports on the medical use of MSCs are for the treatment of injuries of skeletal muscle, cartilages, and ligaments, which are the main causes of reduced performance in equine athletes, leading to important health and economic issues [36]. MSCs have also been used in mares with endometrial diseases [37,38]. The underlying cause of all these pathologies is inflammation, of which PGE2 is a key mediator.

Besides it roles in inflammation, PGE2 is a regulatory molecule involved in self-renewal of human MSCs [25,26]. The blocking of specific EP2 and EP4 receptors using selective antagonists leads to deregulated cell growth of hMSCs in the presence of PGE2 [27]. Cellular migration in both normal and tumour cells might also be governed by EP receptors [39,40,41]. Cell proliferation and vascularisation in several types of cancer is also mediated by the EP2 receptor via increased secretion of vascular endothelial growth factor [8,11,12]. For all these reasons, manipulation of the EP-PGE2 axis could be of use in modern veterinary regenerative medicine.

Here, we edited the EP2 and EP4 receptors in horse adipose MSCs using CRISPR/Cas9 technology. To accomplish this, we designed gRNAs using the website crispr.dbcls.jp, which minimizes mismatches to reduce the risk of off-target modifications [42]. We reached a 30–38% transduction efficiency with lentiviral cassettes also containing GFP reporter genes for ease of visualisation and sorting. Previous studies in horses have shown that lentiviral vectors pseudo typed with VSV-G are able to stably transduce adipose-derived MSCs over numerous passages, demonstrating a positive correlation between the multiplicity of infection (MOI) and the percentage of GFP-expressing cells [43]. Transduction efficiencies ranged from 26 ± 5% for a MOI of 1, up to 68 ± 6% and 83 ± 3%, for MOI values of 5 and 10 respectively. Studies using lentiviral vectors on human bone marrow MSCs reported efficiencies of 80% for a MOI of 10 [44] and 14% for a MOI of 14 [45], suggesting that transduction efficiency is extremely source dependent. Others have reported similar transduction figures for other primary cells, such as human MSCs [46,47,48]. After transduction, the clones reached confluence in approximately 20 days, which is in agreement with de Oliveira et al. [49], who used bovine heterozygous TFAM fibroblasts cells edited by CRISPR/Cas9. The herein reported transduction efficiency and cell recovery did not greatly differ from other MSC reports. Nevertheless, using FACS separation yields a comparative benefit for selection compared to other methods, such as limiting dilution, which is time consuming, of low throughput, and does not necessarily guarantee clonality [37], nor antibiotic selection [38], which could impair cell growth after editing. 

We identified 11 edited clones of EP2 and 10 clones of EP4 after T7EI digestion. This assay has proven powerful for easy detection of mismatched heteroduplex double-stranded DNA in the CRISPR/Cas9 system [50]. Nevertheless, the calculation of the indel frequency is usually affected by non-specific digestion [51]; thus, sequencing of the targeted region is the ultimate test to determine the actual indel frequency. After sequencing analysis of the clones, the number of actual editions dropped to one per receptor gene: one clone with a bi-allelic 9 bp deletion (homozygote KO) in EP2-edited cells and one clone with a 10 bp deletion in one allele (heterozygotic) in EP4-edited cells.

To confirm the indel mutations, we used the ICE analysis (https://ice.synthego.com) [52]. EP2 and EP4 achieved an indel frequency of 94% and 13%, respectively, and the KO scores were 2 and 10, respectively. This web tool provide an alternative means of analysing CRISPR gene editing efficiency using two electropherograms: one from a Cas9-gRNA-treated cell population, and the other from an untreated control or parental cell population. ICE gives an ICE score (an indel percentage), a KO score (proportion of indels that indicates frameshifts), and an *r^2^* regression showing the degree of alignment between the treated and control (parental) cell populations [53].

Functional analysis of cells with edited genes is mandatory for downstream applications in cellular therapy. The immunocytochemistry and PCR analysis showed a decrease in expression of the EP2 and EP4 receptors in aMSCs. These results are in agreement with Choudhary et al. [54], who demonstrated by real-time PCR and immunocytochemistry a decrease in EP2 receptor expression in EP2 receptor KO mice. Our findings are in agreement also with the reports in murine marrow stroma cells in which, the with heterozygotic KO of the EP2 receptor reduced expression of said receptor by 43% only, and there was a persistent effect of PGE2, probably via a compensatory mechanism involving EP4 receptor of for [54]. EP2 and EP4 receptor gene edition has not been previously reported in horse MSCs; thus, this is a new finding. Both EP2 and EP4 receptors were expressed in the horse oviduct in epithelial cells, vascular endothelium, smooth muscle, and serosa [55]. A relatively lower cell density was observed in edited cells, in general, particularly during the processing for immune staining. However, this did not hamper the analysis in any of the assays; neither was statistically significant in Nucblue live staining (not shown). There are conflicting reports about the potential influence of lentiviral transduction on cell viability. Both negative influences [48,56] and no influences [57] of polybrene on cell proliferation have been reported after lentiviral transduction of primary cultures. Polybrene can disrupt the transmembrane potential in some sensitive cells [56]. We cannot rule out that the lower cell density observed in some of the cultures here can be ascribed to the use of polybrene in the transduction step 

The EP2 receptor has been shown to be important to fertility. EP2−/− female mice have reduced fertility, associated with failure of cumulus expansion and fertilisation in vivo [58,59]. EP2 and EP4 gene expression was also observed in murine ocular inflammation, but this function was impaired in EP2 and EP4 knockout mice [60].

As mentioned earlier, PGE2 facilitation of cellular migration has been well established using several normal cells and tumour cells [41] and can potentiate the migratory capacity of MSCs to target tissue [13]. The migration ability towards a chemoattractant is a very desirable property of MSCs [25]. In our study, we found that manipulation of the prostaglandin EP2 receptor resulted in decreased cellular motility and slower wound closure in the first 24 h in the presence of PGE2, which otherwise disappeared at later time points. Yun and colleagues [61] demonstrated that PGE2 induced increased migration of MSC via EP2 receptor, this migration was stronger at 6 h after exposure to PGE2, but attenuated thereafter. In experiments using human blood endothelial cells (HBEC), individual silencing of the EP2, EP3, or EP4 receptor partially decreased the migratory response of HBEC to PGE2 [62]. Migration of HBEC was time-dependent with a maximum response at 8 h of exposure. The migratory response of PGE2 declined after 8 h, and authors concluded that PGE2 stimulated migration of HBEC occurs via cooperation of the EP2, EP3, and EP4 receptor subtypes. 

In human MSCs, overexpression of EP2 alone does not affect the migration of MSCs. However, PGE2-treated MSC-EP2+ almost filled the scratched area, and the width was significantly narrower than that of PGE2-free MSCs [13]. Thus, manipulating the axis of EP-PGE2 might indeed be of use in certain cellular therapies involving PGE2.

Finally, we decided to test if the editing procedure and subsequent selection/expansion affected the surface markers of stemness in the MSCs by analysing their expression in RT-PCR assays. As in native (wild type) adipose MSCs from the laboratory used here, the edited cells kept the same (CD90, CD44, CD166, CD29, CD79a MHCI)^+^/(CD45, MHC II )^−^ phenotype of surface stemness markers. Our findings are essentially in agreement with the accepted phenotype of human MSCs [29], as well as with consensus markers for horse MSCs [25,63,64]. Due to the relatively low cross-reactivity between human and horse antibodies for the markers listed above [65,66], we opted to use RT-qPCR with previously validated primers [30] to analyse the transcript expression of the surface markers in wild-type cells and edited cells.

It has been demonstrated that PGE2 secretion from adult stem cells affects their own proliferation in an autocrine loop mediated by EP2 receptor [1], and so therefore the suppression of the PGE2 axis may influence other properties of MSCs, such as the expression of surface markers. In the mentioned report, expression of surface markers of stemness was not affected either by the use of a direct inhibitor of COX-2 synthesis like celecoxib, nor by blocking mPGES-1 expression using a chemical inhibitor (CAY 10526) in human umbilical cord-MSC. In addition, the expression levels of pluripotency marker genes in the MSC were not significantly altered by COX-2 inhibition. In our research, the surface markers of MSC were also not altered after edition of EP2 or EP4 receptors. Taken together, the inhibition or down-regulation of the COX-2/PGE2 axis seems to not affect the expression levels of selected stem cell marker genes, even though this axis plays a critical role in maintaining self-renewal of MSC.

## 5. Conclusions

We successfully targeted EP2 and EP4 receptor genes in horse adipose MSCs using the CRISPR/Cas9 system. This targeting did not affect the surface marker phenotype of the generated adipose MSCs. Instead, it did affect the initial (early) migration ability of aMSCs/EP2 and significantly lowered the expression of each of the targeted receptors. This opens the possibility of using these mutant cell lines as a model system to elucidate the role of EP2 and EP4 in MSCs and, particularly, as therapeutic tools in equine regenerative medicine.

## Figures and Tables

**Table 1:** Primers sets used for PCR from genomic DNA and the T7EI assay.

Name	Sequence (5’–3’)	Product Size	Position Sequence
PTGER2_sgRNA	Fwd: CACCGTGGTGCTGGCTTCGTACGCG		226/Fwd
	Rev: AAACCGCGTACGAAGCCAGCACCAC		
PTGER2_Ontarget	Fwd: GGAGGACTCGTCTCCCTCTT	393 bp	
	Rev: AGGCTGAAGAAGGTCATGGC		
PTGER4_sgRNA	FwdCACCGGAGACGACCTTCTACACGT		230/Fwd
	Rev: AAACACGTGTAGAAGGTCGTCTCC		
PTGER4_Ontarget	Fwd: GGACACGTAGACGGCAAAGA	358 bp	
	Rev: TGTTCATCTTCGGGGTGGTG		

Fwd = Forward, Rev = Reverse.

**Table 2:** Primers used for relative quantification of the *PTGER2* and *PTGER4* receptors and housekeeping (ACTB).

Acess number	Gene Symbol	Primer Sequence (5’-3’)	Product Size
NM_001127352.1	*PTGER2*	Fw: CATCAGCTCCGTGATGGTCT	294 bp
		Rev: ATCGTGGCCAGGCTGAAGA	
XM_001499068.5	*PTGER4*	Fw: AGCTCCAACCTGCCCAAGAGT	406 bp
		Rev: CATTGGACACGTAGACGGCAAA	
NM_001081838.1	*ACTB*	Fw: GCTCCCAGCACGATGAAGAT	93 bp
		Rev: GGTGGACAATGAGGCCAGAA	
NM_001163856.1	*GAPDH*	Fw: GGGTGGAGCCAAAAGGGTCATCAT	80 bp
		Rev: AGCTTTCTCCAGGCGGCAGGTCAG	
NM_001301217.1	*CD29*	Fw: GTGAGATGTGTCAGACGTGC	216 bp
		Rev: AGAACCAGCAGTCATCCACA	
NM_001085435.2	*CD44*	Fw: TTCATAGAAGGGCACGTGGT	237 bp
		Rev: GCCTTTCTTGGTGTAGCGAG	
XM_001503225.4	*CD90*	Fw: TCTCCTGCTGACAGTCTTGC	242 bp
		Rev: GGACCTTGATGTTGTACTTGC	
XM_001503380.6	*CD166*	Fw: GCAGAAAACCAGCTGGAGAG	159 bp
		Rev: AGCGAGGAGTAGACCAACGA	
NM_001309162.1	*CD45*	Fw: CTCCTCATTCACTGCAGAGA	163 bp
		Rev: GGTACTGCTCAAATGTGGGA	
NM_001082508.2	*MHC I*	Fw: TTCATCTCCGTCGGCTACGTG	334 bp
		Rev: AGGAGCGCAGGTCCTCGTT	
NM_001142816.1	*MHC II*	Fw: AGCGGCGAGTTGAACCTACAGT	172 bp
		Rev: CGGATCAGACCTGTGGAGATGA	

## Supplementary Materials

The following are available online at https://www.mdpi.com/2076-2615/10/6/1078/s1, Table S1: Primers used for cloning of equine EP2 and EP4 receptors.
