# Supplementary material for: Edition of Prostaglandin E2 Receptors EP2 and EP4 by CRISPR/Cas9 Technology in Equine Adipose Mesenchymal Stem Cells
Source: Animals (Basel). 2020 Jun 23;10(6):1078. doi: 10.3390/ani10061078 (PMC7341266; doi:10.3390/ani10061078)
Supplement: Supplementary file 1 [file animals-10-01078-s001.pdf]

Supplementary File 1

Table S1. Primers used for cloning of equine EP2 and EP4 receptors.

Acession Number	Gene Symbol	PrimerSequence (5'–3′)	Product Size
NM_001127352.1	*PTGER2*	Fw: CATCAGCTCCGTGATGGTCT	294 bp
		Rev: ATCGTGGCCAGGCTGAAGA	
		Fw: TTCGCCTTCGCCATGACCTT	247 bp
		Rev: TGCGTCCATGCCGGATGAAA	
		Fw: TGGTGTTTCATCCGGCATGG	359 bp
		Rev: GACAGAAACCTAAGGGCTTGGA	
XM_001499068.5	*PTGER4*	Fw: GGATGAAGCCTTTTCCCGAAGA	256 bp
		Rev: TCCAGATGCTAACTCAGCACT	
		Fw: AGCTCCAACCTGCCCAAGAGT	406 bp
		Rev: CATTGGACACGTAGACGGCAAA	
		Fw: TCAACCACGCCTACTTCTACAG	481 bp
		Rev: GAGGTGGCGATGAGTAAGATGA	
		Fw: AGTTCAGAGCAAGACGTCAGCA	263 bp
		Rev: ATCTCCTTCAGTTCCCGGGAAA	
		Fw: TCCTTCCTTTCCCGGGAACT	420 bp
		Rev: AGCCCTTCTGAGCACAGCTAAA	
